# Romiplostim for cancer treatment-induced thrombocytopenia in acute promyelocytic leukemia: a case report

**DOI:** 10.3389/fonc.2026.1915786

**Published:** 2026-08-26

**Authors:** Xiaoye Yuan, Qixin Guo, Zewen Zhang

**Affiliations:** Department of Hematology, The Second Affiliated Hospital, Shantou University Medical College, Shantou, Guangdong, China

**Keywords:** acute promyelocytic leukemia, arsenic trioxide, cancer treatment-induced thrombocytopenia, romiplostim, thrombopoietin receptor agonists, TPO-RAs

## Abstract

**Background:**

All-trans retinoic acid (ATRA) combined with arsenic trioxide (ATO) is a first-line treatment regimen for acute promyelocytic leukemia (APL), with generally milder myelosuppressive effects compared with conventional chemotherapy. Although standard-dose ATO is not typically considered a major risk factor for cancer treatment-induced thrombocytopenia (CTIT), severe and persistent thrombocytopenia may still occur during ATO maintenance therapy, and its clinical characteristics and therapeutic strategies remain unclear.

**Case presentation:**

A 60-year-old female patient with APL developed refractory grade 3–4 thrombocytopenia during ATO maintenance therapy, with no significant response to recombinant human thrombopoietin or hetrombopag treatment (platelet nadir: 8 × 10^9^/L; duration of platelet count <50 × 10^9^/L: 8 weeks). Subsequently, romiplostim (250 μg/week) was administered, resulting in platelet recovery to 151 × 10^9^/L within 3 weeks, accompanied by restoration of megakaryocyte numbers and reappearance of platelet-producing megakaryocytes in the bone marrow. The patient subsequently resumed maintenance therapy and completed the remaining treatment cycles with romiplostim support, maintaining continuous molecular remission during follow-up.

**Conclusion:**

This case suggests that refractory CTIT characterized by predominant megakaryocytic involvement may occur during ATO maintenance therapy, with clinical features potentially differing from those of conventional cytotoxic chemotherapy-related CTIT. Romiplostim may promote the recovery of residual megakaryopoietic capacity and provide a potential therapeutic option for such patients. However, its precise mechanism of action and the patient populations most likely to benefit require further investigation.

## 1 Introduction

Acute promyelocytic leukemia (APL) is a distinct subtype of acute myeloid leukemia characterized by the presence of the PML-RARA fusion gene resulting from the reciprocal translocation t(15;17) (1). All-trans retinoic acid (ATRA) combined with arsenic trioxide (ATO) has become the standard treatment regimen for patients with low- and intermediate-risk APL, achieving higher complete remission rates and favorable long-term survival outcomes compared with conventional ATRA-based chemotherapy regimen (2, 3). However, ATO treatment may still be associated with adverse events, including cardiotoxicity, hepatotoxicity, and differentiation syndrome. Although ATO generally causes less myelosuppression than conventional chemotherapy, hematological toxicities, including neutropenia and thrombocytopenia, may still occur, particularly during prolonged treatment (4–6).

Cancer treatment-induced thrombocytopenia (CTIT) is a common complication during anticancer therapy and may increase the risks of bleeding, treatment interruption, and dose modification (7). Compared with conventional cytotoxic chemotherapy, standard-dose ATO is generally not considered a major cause of severe thrombocytopenia. Although thrombocytopenia during ATO treatment has been reported, severe and persistent CTIT occurring during ATO maintenance therapy appears to be uncommon, and its incidence and clinical characteristics remain unclear. Unlike chemotherapy-associated CTIT, which typically involves extensive bone marrow suppression, thrombocytopenia associated with non-cytotoxic therapies may involve distinct abnormalities in hematopoietic regulation.

Thrombopoietin receptor agonists (TPO-RAs) have become potential therapeutic options for refractory CTIT by enhancing megakaryopoiesis and platelet production. Previous studies have demonstrated that TPO-RAs achieve high platelet response rates and an acceptable safety profile in patients with CTIT associated with solid tumors and hematological malignancies (8, 9). Although no TPO-RA has been specifically approved for the treatment of CTIT, current clinical guidelines allow consideration of off-label romiplostim use in selected patients who require continuation of anticancer therapy. However, the available evidence supporting the use of romiplostim for CTIT is primarily derived from patients receiving cytotoxic chemotherapy, and data regarding its application in ATO-associated CTIT remain extremely limited.

Here, we report a case of an APL patient who developed prolonged refractory CTIT characterized by selective megakaryocytic suppression during ATO maintenance therapy. The patient showed no response to recombinant human thrombopoietin or hetrombopag but achieved sustained platelet recovery after romiplostim treatment, allowing continuation of maintenance therapy and maintenance of molecular remission. This case provides clinical observations regarding the management of refractory thrombocytopenia in the specific context of ATO-associated CTIT.

## 2 Case presentation

A 60-year-old female patient was admitted on February 22, 2022, due to pancytopenia identified during a routine examination. The patient had no significant clinical symptoms prior to admission. Physical examination revealed no superficial lymphadenopathy, sternal tenderness, or hepatosplenomegaly.

**Figure 1:**
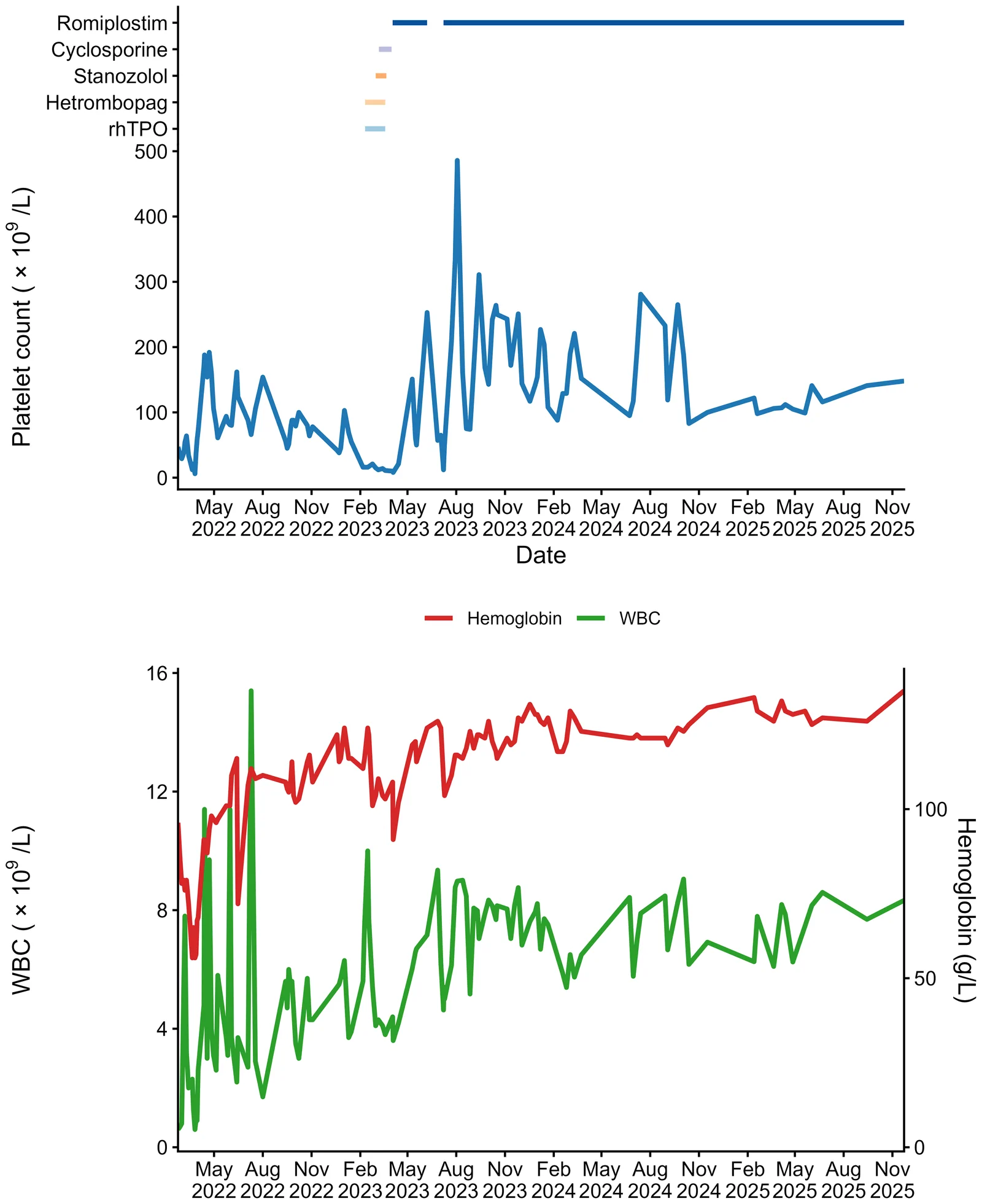
Dynamic changes in blood cell counts and sequential thrombopoietic interventions during treatment. The figure illustrates the changes in platelet counts during different thrombopoietic interventions, together with the concurrent dynamics of white blood cell and hemoglobin levels. During treatment with rhTPO combined with hetrombopag, followed by the addition of stanozolol and cyclosporine, the platelet count remained persistently below 50 × 10^9^/L without significant improvement. After initiation of romiplostim, the platelet count rapidly increased and returned to the normal range. Throughout the treatment course, white blood cell and hemoglobin levels remained relatively stable, suggesting that the patient presented predominantly with isolated persistent thrombocytopenia rather than generalized bone marrow suppression.

Initial laboratory examinations revealed pancytopenia, including a white blood cell count of 0.6 × 10^9^/L, hemoglobin level of 96 g/L, and platelet count of 47 × 10^9^/L (**Figure 1**). Coagulation tests showed a mildly prolonged thrombin time (TT) of 18.2 seconds, an elevated D-dimer level of 0.96 μg/mL, and a positive protamine sulfate precipitation test. However, prothrombin time (PT), activated partial thromboplastin time (APTT), and fibrinogen levels were within the normal ranges, and no evidence of active bleeding was observed.

**Figure 2:**
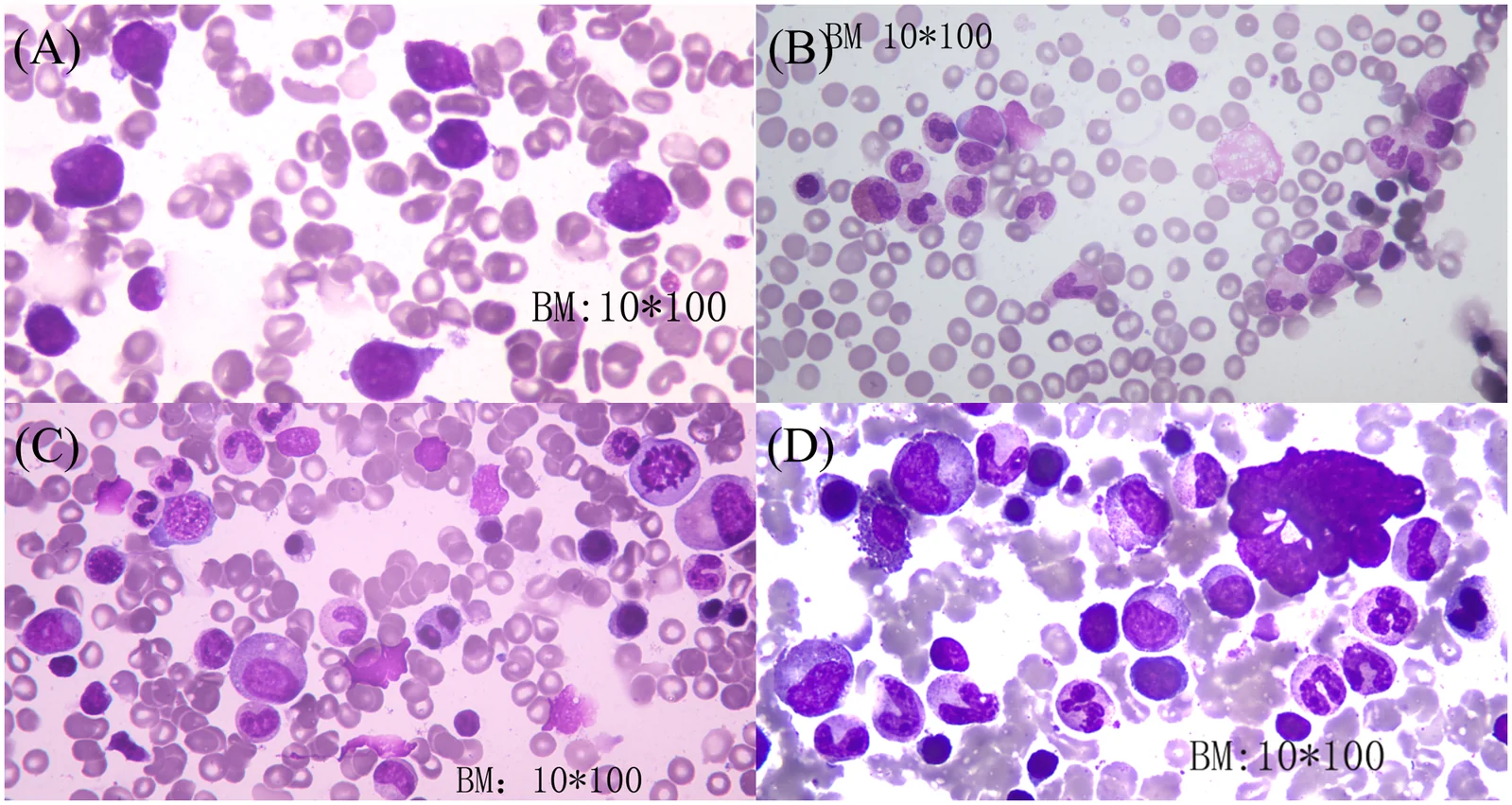
Morphological evolution of bone marrow in an APL patient from diagnosis to remission. **(A)** At diagnosis (February 2022): The bone marrow was hypercellular with abundant abnormal promyelocytes, and no megakaryocytes were observed on the entire slide. **(B)** Early maintenance therapy (January 2023): The bone marrow was normocellular with no abnormal promyelocytes observed. A total of 22 megakaryocytes were counted on the entire slide, with platelets distributed singly or in small clusters. **(C)** During maintenance therapy (February 2023): The bone marrow was normocellular with no abnormal promyelocytes observed. A significant reduction in megakaryocytes was noted, with only one granular megakaryocyte found across two slides, accompanied by thrombocytopenia. **(D)** Post-treatment follow-up (September 2024): After romiplostim therapy and a full course of maintenance treatment, the bone marrow remained normocellular and exhibited active trilineage hematopoiesis. All slides were stained with Wright-Giemsa.

Bone marrow aspiration revealed markedly active marrow proliferation, with approximately 60% of cells consisting of abnormal promyelocytes exhibiting typical morphological features of APL. No megakaryocytes were observed throughout the entire bone marrow smear, accompanied by thrombocytopenia (**Figure 2A**). Flow cytometric analysis showed that the abnormal cells expressed CD117, CD33, and CD13, while lacking CD34 and HLA-DR expression. Cytogenetic analysis identified the translocation t(15;17)(q24;q21), and molecular testing confirmed the presence of the PML::RARA fusion transcript. Based on the findings from bone marrow morphology, immunophenotyping, cytogenetics, and molecular analysis, the patient was diagnosed with APL (FAB M3 subtype).

The patient subsequently received induction therapy with ATRA combined with ATO and completed three cycles of consolidation therapy, during which hematological remission was achieved. Transient thrombocytopenia occurred during the consolidation phase and recovered after treatment with recombinant human thrombopoietin (rhTPO). Follow-up bone marrow examination demonstrated preserved megakaryopoiesis (>50 megakaryocytes per smear), suggesting recovery of hematopoietic function after treatment.

The patient initiated ATO maintenance therapy on day 174 (August 15, 2022). During the early stage of maintenance therapy, the patient gradually developed mild-to-moderate thrombocytopenia. Serial bone marrow examinations during this period demonstrated a progressive decline in megakaryocyte numbers: approximately 32 megakaryocytes per smear were observed during the first maintenance cycle, decreasing to 22 per smear during the second maintenance cycle (**Figure 2B**).

On day 349 (February 6, 2023), the patient developed oral mucosal blood crusting, with a platelet count decreasing to 8× 10⁹/L, indicating grade 3–4 thrombocytopenia (**Figure 1**). At the same time, coagulation tests showed that PT, APTT, fibrinogen, and D-dimer levels were within the normal ranges, with only mildly prolonged TT (17.2 s). No evidence supporting persistent coagulation activation or consumptive coagulopathy was identified. Given the severe thrombocytopenia, ATO therapy was temporarily discontinued, and the patient underwent further evaluation and treatment.

On day 353 (February 10, 2023), the patient initiated treatment with rhTPO (15,000 U/day) combined with hetrombopag (2.5 mg/day), which was continued for 38 days; however, no significant improvement in platelet counts was observed. Subsequently, the patient received glucocorticoids, stanozolol, and cyclosporine. Due to diabetes-related considerations, glucocorticoid therapy was administered for only 1 day, while stanozolol (2 mg/day) and cyclosporine (100 mg/day) were administered for 20 and 25 days, respectively. Despite these treatments, the platelet count remained below 50× 10⁹/L for approximately 8 weeks (**Figure 1**).

Bone marrow examination during this period demonstrated a further reduction in megakaryocytes, with only one granular megakaryocyte identified in two bone marrow smears (**Figure 2C**). In contrast, granulopoiesis and erythropoiesis were relatively preserved, without evidence of extensive bone marrow failure. Flow cytometry revealed no abnormal promyelocyte population, and neither bone marrow morphology nor PML::RARA testing indicated APL relapse. To evaluate the possibility of immune-mediated thrombocytopenia, the patient underwent further autoimmune assessments, including antiphospholipid antibody testing (anticardiolipin antibodies, anti-β2 glycoprotein I antibodies, and lupus anticoagulant), serum immunoglobulin levels, and complement levels, all of which showed no abnormalities.

Considering the patient’s disease status, temporal relationship with treatment, laboratory findings, and serial changes in bone marrow morphology, the patient was diagnosed with refractory CTIT associated with ATO maintenance therapy, primarily characterized by persistent and selective impairment of megakaryopoiesis.

On day 405 (April 3, 2023), the patient developed tongue bleeding and an approximately 0.5-cm ecchymosis, with a platelet count of 10× 10⁹/L (**Figure 1**). Given the persistent severe thrombocytopenia, risk of active bleeding, and lack of significant response to previous treatments including rhTPO, hetrombopag, and immunomodulatory therapy, romiplostim was initiated as salvage therapy.

Romiplostim was initiated at an initial dose of 250 μg/week, which was subsequently maintained at the same dose. Following treatment, the patient’s platelet count increased rapidly, reaching 151× 10⁹/L on day 442 (May 10, 2023) (**Figure 1**). Bone marrow re-examination on day 451 (May 19, 2023) demonstrated recovery of megakaryopoiesis and reappearance of platelet-producing megakaryocytes.

The patient subsequently continued romiplostim support. On day 472 (June 7, 2023), the platelet count increased to 253 × 10^9^/L, and romiplostim treatment was therefore discontinued. On day 503 (July 8, 2023), the patient developed recurrent oral mucosal bleeding, with the platelet count decreasing to 12 × 10^9^/L. After platelet transfusion and re-initiation of romiplostim treatment, the platelet count recovered to 52× 10⁹/L on day 505 (July 10, 2023) (**Figure 1**).

Following recovery of platelet counts, on day 491 (June 26, 2023), the patient initiated subsequent APL maintenance therapy with Compound Huangdai Tablet combined with all-trans retinoic acid (ATRA) and completed the remaining maintenance cycles with romiplostim support. During follow-up until July 2025, the patient remained in continuous molecular remission, with no evidence of disease relapse based on bone marrow examination, flow cytometry, and PML::RARA fusion gene testing (**Figure 2D**).

## 3 Discussion

This case report describes an APL patient who developed refractory treatment-related thrombocytopenia during ATO maintenance therapy and achieved sustained platelet recovery after romiplostim treatment. Several features of this case are noteworthy. First, the patient developed persistent severe thrombocytopenia during the maintenance phase of ATO, a differentiation therapy agent, without exposure to conventional cytotoxic chemotherapy. Second, serial bone marrow morphological examinations demonstrated a progressive decline in megakaryocyte numbers across treatment cycles, followed by recovery after romiplostim treatment, suggesting that the thrombocytopenia may have been associated with impaired megakaryopoiesis. In addition, the patient showed a clear response to romiplostim after failure of multiple platelet-raising therapies, allowing completion of subsequent treatment and maintenance of molecular remission.

Currently, the clinical evidence supporting romiplostim for the treatment of CTIT is primarily derived from patients receiving cytotoxic chemotherapy. The phase II study by Soff et al. and the recent phase III RECITE trial both demonstrated that romiplostim could promote platelet recovery in a subset of patients with refractory CTIT and reduce treatment delays caused by thrombocytopenia (10, 11). However, these studies mainly enrolled patients with solid tumors, and data from patients with APL receiving ATO treatment are lacking. Therefore, the findings from studies of conventional chemotherapy-related CTIT cannot be directly extrapolated to ATO-associated thrombocytopenia. This case suggests that CTIT occurring during ATO maintenance therapy may represent a clinical phenotype distinct from classical cytotoxic chemotherapy-related CTIT, characterized by features including prolonged drug exposure, selective megakaryocytic involvement, and relative preservation of other hematopoietic lineages.

Before diagnosing ATO-associated CTIT, we evaluated other potential causes of thrombocytopenia. At the time of the platelet nadir, no evidence of persistent coagulation activation or consumptive coagulopathy was identified, reducing the likelihood of disseminated intravascular coagulation (DIC) as a cause of thrombocytopenia. Flow cytometry, bone marrow morphology, and PML::RARA testing did not indicate APL relapse. Bone marrow examination demonstrated preserved nucleated cell proliferation without evidence of extensive bone marrow failure. In addition, autoimmune-related examinations revealed no significant abnormalities. However, it should be noted that anti-platelet glycoprotein IIb/IIIa antibodies were not assessed in this case; therefore, antibody-mediated immune thrombocytopenia cannot be completely excluded. Nevertheless, the patient showed no significant response to treatments such as cyclosporine, while serial bone marrow examinations demonstrated a progressive decline in megakaryocyte numbers (from 22 to 1), rather than the increased megakaryopoiesis commonly observed in immune thrombocytopenia. Therefore, considering the treatment timeline, dynamic changes in bone marrow findings, and therapeutic responses, the overall evidence supports treatment-related thrombocytopenia primarily characterized by impaired megakaryopoiesis.

Reviewing the patient’s entire treatment course, the reduction in megakaryocytes at initial diagnosis may have been associated with APL-related bone marrow abnormalities. However, after induction and consolidation therapy, the patient achieved hematopoietic recovery, with normalization of platelet counts. Therefore, the persistent thrombocytopenia that recurred during the ATO maintenance phase does not appear to reflect the previous disease status or residual bone marrow injury after treatment but rather may represent a newly developed hematopoietic abnormality in the context of prolonged ATO exposure. Nevertheless, as this is a single-case observation, a direct causal relationship between ATO exposure and megakaryocytic impairment cannot currently be established.

The potential mechanisms underlying ATO-associated CTIT remain unclear. Previous studies have suggested that the effects of ATO on the hematopoietic system may depend on drug exposure duration, dosage, and the stage of cellular differentiation. Short-term ATO exposure has been reported to promote terminal differentiation of mature megakaryocytes in some experimental models, whereas prolonged or repeated exposure may affect hematopoietic progenitor cell maintenance and lineage differentiation (12–16). Therefore, the pro-differentiation effects observed after short-term exposure are not necessarily inconsistent with the development of hematopoietic homeostasis disturbances following long-term exposure.

It should be emphasized that there is currently no direct human evidence demonstrating that ATO has a specific toxic effect on megakaryocytes. Therefore, the reduction in megakaryocytes observed in this case is more likely to reflect the impact of long-term ATO exposure on the hematopoietic regulatory network rather than a confirmed cell-specific cytotoxic effect.

The predominant involvement of megakaryocytes with relative preservation of erythropoiesis observed in this case may be attributable to the intrinsic heterogeneity of hematopoietic progenitor populations. Although megakaryocytes and erythrocytes both originate from common megakaryocyte–erythroid progenitors (MEPs), recent studies have demonstrated that MEPs are not a completely homogeneous population of bipotent cells but rather comprise subpopulations with distinct lineage biases (17). Therefore, impairment at the level of common progenitors does not necessarily result in equivalent damage across all downstream lineages. Different MEP subsets may exhibit variable sensitivity to drug exposure, which may explain the marked impairment of megakaryopoiesis with relative preservation of erythroid and myeloid lineages observed in this case.

In addition, previous studies have suggested that ATO may affect other hematopoietic lineages, including erythroid-related processes (18). However, these studies were mainly derived from experimental models or limited clinical observations and cannot predict the occurrence of multilineage hematopoietic suppression in all patients. A long-term follow-up study by Santinelli et al. demonstrated that APL patients receiving ATRA combined with ATO therapy may develop myelodysplastic changes in the bone marrow, with relatively prominent abnormalities in the megakaryocytic lineage (19). Although this study did not establish that ATO causes megakaryocyte-specific injury, its findings provide some clinical support for the possibility of persistent megakaryocytic abnormalities following long-term ATO exposure.

Currently, thrombopoietin receptor agonists (TPO-RAs) have not been formally approved for the treatment of CTIT; however, previous studies have supported their potential value in patients with refractory CTIT who require continuation of anticancer therapy. Studies by Soff et al. and Al-Samkari et al. demonstrated that romiplostim achieved relatively high response rates in patients with CTIT (10, 20). The RECITE trial further supported that romiplostim could significantly reduce chemotherapy dose delays and modifications (11). However, these studies primarily focused on CTIT associated with cytotoxic chemotherapy; therefore, whether their efficacy and mechanisms of action are applicable to ATO-associated CTIT requires further investigation.

In this case, the patient continued to experience persistent thrombocytopenia and required transfusion support despite treatment with rhTPO and hetrombopag. In contrast, romiplostim treatment resulted in rapid platelet recovery, accompanied by restoration of megakaryocyte numbers on bone marrow examination. These findings suggest that, even in the presence of marked megakaryocyte reduction, a certain degree of residual megakaryopoietic potential may be preserved, and activation of the TPO/MPL signaling pathway may promote the recovery of residual hematopoietic capacity.

Romiplostim and hetrombopag both promote platelet production through activation of MPL; however, their mechanisms of action differ. As a peptide-based thrombopoietin receptor agonist, romiplostim directly binds to MPL and induces receptor activation (21). In contrast, hetrombopag is an oral small-molecule thrombopoietin receptor agonist that induces receptor conformational changes by binding to a specific site near the transmembrane region of MPL, thereby activating downstream signaling pathways (22). Therefore, the two agents may produce different therapeutic responses in the context of different types of hematopoietic injury. Recent studies have suggested that the response to thrombopoietin receptor agonists (TPO-RAs) may be influenced by factors including disease context, the hematopoietic microenvironment, and residual megakaryocyte reserve. Their efficacy is not solely determined by the drugs themselves but is closely associated with the patient’s baseline hematopoietic status (23).

## 4 Strengths and limitations

The major strength of this case is the longitudinal characterization of ATO-associated thrombocytopenia through serial platelet kinetics and bone marrow morphological assessments. The dynamic changes in megakaryocyte numbers before and after romiplostim treatment provide clinical evidence for understanding the potential mechanisms underlying this rare complication.

Several limitations should also be acknowledged. First, as a single-case report, this study cannot establish a direct causal relationship between ATO exposure and thrombocytopenia. Second, although comprehensive evaluations were performed to exclude alternative causes of thrombocytopenia, including APL relapse, coagulation abnormalities, and autoimmune disorders, anti-platelet glycoprotein IIb/IIIa antibodies were not assessed; therefore, an immune-mediated mechanism cannot be completely excluded. Third, the patient received multiple interventions, including ATO discontinuation, rhTPO, hetrombopag, immunomodulatory therapy, and romiplostim, and the potential contribution of these treatments to platelet recovery cannot be entirely ruled out. Finally, further mechanistic investigations, such as analyses of thrombopoietin levels, megakaryocyte progenitor populations, and hematopoietic cell function, were not available, and future studies are required to clarify the biological mechanisms underlying ATO-associated CTIT.

## 5 Patient perspective

During ATO maintenance therapy, the patient reported that persistent thrombocytopenia and recurrent bleeding events significantly affected her daily life and caused considerable concern, particularly because severe thrombocytopenia resulted in treatment interruption, frequent monitoring, and multiple adjustments of the therapeutic regimen. After receiving romiplostim treatment, the patient’s platelet count recovered and remained stable, allowing reinitiation and completion of the remaining maintenance therapy. The patient reported that improvement in platelet counts and reduced bleeding risk relieved concerns related to treatment interruption and future bleeding events, and she expressed satisfaction with completing the planned therapeutic regimen.

## 6 Conclusion

In conclusion, this case suggests that megakaryopoietic impairment associated with long-term ATO exposure may represent a clinical phenotype distinct from conventional chemotherapy-related CTIT. In this specific context, romiplostim may provide a therapeutic option for patients with refractory thrombocytopenia. Further studies are needed to elucidate the mechanisms underlying ATO-associated CTIT and to identify patient populations most likely to benefit from different types of thrombopoietin receptor agonists in various treatment-related thrombocytopenic conditions.

## Data Availability

The raw data supporting the conclusions of this article will be made available by the authors, without undue reservation.
